# Clinical outcomes and anti-inflammatory mechanisms of nucleus basalis of Meynert deep brain stimulation in Alzheimer’s disease

**DOI:** 10.3389/fneur.2026.1773910

**Published:** 2026-04-22

**Authors:** Yingchuan Chen, Tingting Du, Tenghong Lian, Yin Jiang, Tianshuo Yuan, Jing Li, Fangang Meng, Anchao Yang, Wei Zhang, Jianguo Zhang

**Affiliations:** 1 Department of Neurosurgery, Beijing Tiantan Hospital, Capital Medical University, Beijing, China; 2 Department of Functional Neurosurgery, Beijing Neurosurgical Institute, Capital Medical University, Beijing, China; 3 Department of Neurology, Beijing Tiantan Hospital, Capital Medical University, Beijing, China; 4 Beijing Key Laboratory of Neurostimulation, Beijing, China

**Keywords:** Alzheimer’s disease (AD), cognitive impairment, disease severity, inflammation, nucleus basalis of Meynert deep brain stimulation (NBM-DBS)

## Abstract

**Aim:**

Deep brain stimulation of the nucleus basalis of Meynert (NBM-DBS) represents an emerging therapeutic strategy for Alzheimer’s disease (AD), yet clinical outcomes have been inconsistent and its mechanistic underpinnings are not fully elucidated. This study aimed to assess the cognitive and psychobehavioral effects of NBM-DBS and to explore its potential impact on systemic inflammatory markers.

**Methods:**

In this open-label trial, nine individuals with moderate-to-severe AD underwent bilateral NBM-DBS. Six participants (four with moderate and two with severe AD) completed the full 12-month protocol, which included serial neuropsychiatric assessments and serum cytokine profiling.

**Results:**

Stratification by baseline disease severity revealed divergent cognitive trajectories. Patients with moderate AD (CDR = 2) maintained their preoperative performance on the Montreal Cognitive Assessment (MoCA) and Boston Naming Test (BNT) over the 12-month follow-up. In contrast, patients with severe AD (CDR = 3) experienced significant decline on these measures. Serum analyses demonstrated a significant immunomodulatory effect, characterized by elevated levels of the anti-inflammatory cytokines IL-10 and IL-27, and reduced levels of the pro-inflammatory chemokines CXCL10 and RANTES at the 12-month timepoint.

**Conclusion:**

Our findings indicate that NBM-DBS may be associated with stabilization of cognitive function in patients with moderate AD, potentially through the modulation of inflammation. The therapeutic benefit appears to be more pronounced in the moderate stage of the disease.

## 1 Introduction

Alzheimer’s disease (AD) stands as the most common neurodegenerative disorder, with its prevalence escalating in tandem with global population aging (1). Occurrent estimates indicate approximately 55 million people are living with dementia worldwide, a number projected to rise to 152 million by 2050, with the most substantial increases expected in developing nations (2). In its early stages, AD manifests with symptoms such as short-term memory loss, a decline in executive functions, difficulties in word-finding, and visuospatial dysfunction (1). In the severe or late-stage of AD, there is progressive deterioration of both functional and cognitive abilities. Patients may reach a point where they are unable to recognize their family members, become bedridden, and experience difficulties in swallowing and urination. Eventually, these complications can lead to death (1, 3).

In the landscape of limited disease-modifying pharmacotherapies, neuromodulation approaches such as deep brain stimulation of the nucleus basalis of Meynert (NBM-DBS) have garnered significant interest. The NBM serves as the principal source of cholinergic innervation to the cerebral cortex, a system fundamentally involved in attention, learning, and memory processes that undergoes profound degeneration in AD (4). Initial trials have suggested that NBM-DBS is well-tolerated, without reports of severe or persistent stimulation-related adverse events (5). However, its therapeutic efficacy has varied across studies. For instance, Kuhn et al. (4) documented stabilized cognitive performance and enhanced cerebral glucose metabolism in a subset of patients with mild-to-moderate AD (4), whereas our prior investigation in an advanced cohort did not find significant cognitive improvement at 12 months (6). This discrepancy underscores the critical need to identify patient subgroups most likely to respond to this invasive intervention.

Chronic inflammation has been firmly established as a core pathological driver in AD (7). The complex interplay between activated microglia, astrocytes, and neurons sustains a cytotoxic milieu that exacerbates neurodegeneration. Consequently, profiling inflammatory biomarkers offers a valuable perspective on disease activity and treatment response.

Building upon this foundation, the current study was designed to evaluate the cognitive and psychobehavioral outcomes of NBM-DBS in patients stratified by disease severity and to investigate its influence on systemic inflammatory signaling through comprehensive serum cytokine and chemokine analysis.

## 2 Methods and materials

### 2.1 Study participants and ethical considerations

The study protocol received approval from the Ethics Committee of Beijing Tiantan Hospital (KY 2018-051-02) and was conducted in accordance with the Declaration of Helsinki. Written informed consent was secured from all patients or their legal guardians.

Nine patients (age range: 59–80 years) who fulfilled the National Institute of Neurological and Communicative Disorders and Stroke and the Alzheimer’s Disease and Related Disorders Association (NINCDS-ADRDA) criteria for probable AD were enrolled (8). All participants had a diagnosis of moderate-to-severe dementia, corroborated by Mini-Mental State Examination (MMSE) scores between 2 and 16 (9, 10). Key exclusion criteria encompassed mild AD (MMSE > 20), suicidal ideation, history of intracranial surgery, and contraindications for anesthesia, MRI, or PET.

### 2.2 Electrode implantation surgery and programming

Bilateral DBS electrodes (L301C; PINS Medical Co., Ltd., Beijing) were implanted stereotactically into the posterior Ch4p subregion of the NBM. The surgical target was localized 7 mm posterior to the anterior commissure (AC), 7 mm inferior to the AC-PC line, and 25 mm lateral to the midline, guided by T2-weighted MRI and a Leksell frame (Elekta Instrument AB). Postoperative MRI and CT scans confirmed lead positioning and ruled out procedural complications such as hemorrhage (11, 12). The implanted DBS system is documented as compatible with 3-T MRI (13). Stimulation commenced 1 month after surgery using parameters determined based on previous clinical studies (20 Hz, 2.0–3.0 V, and 90-μs pulse width) (4, 14).

### 2.3 Clinical assessments

Global dementia severity was assessed at baseline using the Clinical Dementia Rating (CDR) scale. A comprehensive battery of neuropsychiatric tests was administered preoperatively and at 1, 3, 6, and 12 months post-surgery. This battery included the MMSE, Alzheimer’s Disease Assessment Scale-Cognitive subscale (ADAS-Cog), Montreal Cognitive Assessment (MoCA), Boston Naming Test (BNT), Modified Apathy Estimate Scale (MAES), Neuropsychiatric Inventory (NPI), Cohen-Mansfield Agitation Inventory (CMAI), and Activity of Daily Living Scale (ADL).

### 2.4 Sample collection and cytokine and chemokine analysis

Peripheral venous blood samples were collected preoperatively and at the 12-month follow-up. Serum was isolated via centrifugation and stored at −80 °C. Concentrations of 34 cytokines and chemokines were quantified using the Cytokine and Chemokine 34-Plex Human ProcartaPlex kit (Invitrogen) on a Bio-Plex200 platform (Bio-Rad), adhering to the manufacturer’s instructions.

### 2.5 Statistical analysis

Data are expressed as mean ± standard deviation Statistical analyses were conducted using GraphPad Prism (10.1, GraphPad Software, Inc., San Diego, CA, United States). Longitudinal clinical data were analyzed by one-way repeated-measures ANOVA (RM-ANOVA) with LSD *post hoc* tests for pairwise comparisons. Changes in inflammatory marker concentrations from baseline to 12 months were evaluated using paired-sample *t*-tests.

## 3 Results

### 3.1 Patient cohort and cognitive outcomes

**Figure 1:**
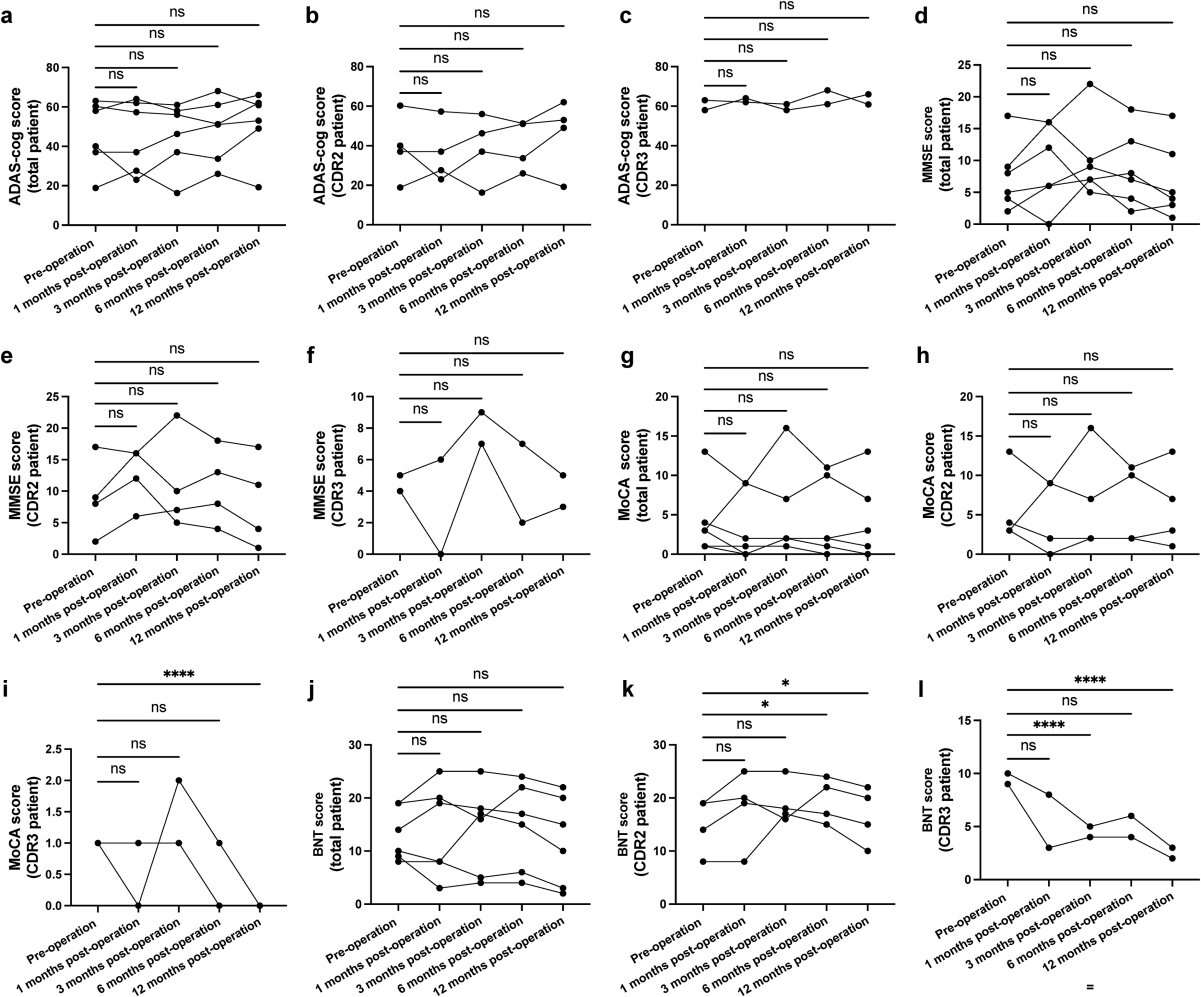
Cognitive evaluation results at baseline and over a 1-year follow-up period during NBM-DBS for the full AD patient cohort and subgroups stratified by Clinical Dementia Rating (CDR) score. **(a–c)** No significant change was found in ASAD-cog score for the entire group [**a**; *F*_(2.009, 10.04)_ = 1.293, *p* = 0.3168], patients with moderate AD [**b**; *F*_(1.838, 5.514)_ = 1.091, *p* = 0.3924], or patients with severe AD [**c**; *F*_(1.000, 1.000)_ = 0.7032, *p* = 0.5558]. **(d–f)** Similarly, there was no significant change in Mini Mental State Examination (MMSE) score during treatment compared to baseline. **(g–i)** Montreal Cognitive Assessment (MoCA) score did not change significantly during treatment for the entire cohort [**g**; *F*_(1.746, 8.730)_ = 0.5822, *p* = 0.5572] or the moderate disease subgroup [**h**; *F*_(1.579, 4.737)_ = 0.3484, *p* = 0.6768], but decreased in the severe disease subgroup [**i**; *F*_(1.000, 1.000)_ = 1.857, *p* = 0.4030]. **(j–l)** Boston Naming Test (BNT) scores also did not change for the entire cohort [**j**, *F*_(2.164, 10.82)_ = 0.7762, *p* = 0.4936], but increased at 6 and 12 months post-operation in the moderate subgroup [**k**; *F*_(1.709, 5.128)_ = 2.010, *p* = 0.2250] and declined in the severe subgroup [**l**; *F*_(1.000, 1.000)_ = 8.733, *p* = 0.2077]. PD-DBS group. *p < 0.05; **p < 0.01; ***p < 0.001, ****p < 0.0001; ns, not significant.

From the initial enrollment of nine patients, six completed the full 12-month follow-up; three were lost primarily due to constraints related to the COVID-19 pandemic (Table 1; Patient 1, 6, and 8 were excluded from the following analyses; Supplementary Figure 1). For the entire cohort, no statistically significant changes were observed in ADAS-Cog, MMSE, MoCA, or BNT scores across the follow-up period (all *p* > 0.05) (Figures 1a–f). Stratification based on baseline CDR scores, however, revealed distinct clinical trajectories. In the moderate AD subgroup (CDR = 2), scores on the MoCA and BNT were maintained at the 12-month assessment. Conversely, the severe AD subgroup (CDR = 3) exhibited a significant decline in MoCA performance and a downward trend in BNT scores (Figures 1g–l).

**Table 1:** Summary of patient demographics and current treatments.

Patients	Age	Sex	Medication (daily dose)
Pat1	80	Male	Memantine (10 mg); Donepezil (5 mg); Exelon (3 mg)
Pat2	61	Female	Donepezil (5 mg)
Pat3	67	Male	Donepezil (10 mg); Memantine (20 mg); Aniracetam (0.6 g)
Pat4	66	Male	Donepezil (10 mg); Memantine (20 mg)
Pat5	78	Male	/
Pat6	67	Female	Donepezil (5 mg); Memantine (2.5 mg)
Pat7	70	Male	Donepezil (10 mg)
Pat8	59	Female	Donepezil (10 mg); Huperzine A (0.1 mg); Oxiracetam (0.4 g)
Pat9	65	Male	Donepezil (10 mg); Memantine (5 mg)

To contextualize these observations, we compared our results with natural history data from the National Alzheimer’s Coordinating Center (NACC^1^) database. As anticipated, NACC patients with a CDR of 2 demonstrated significant cognitive decline over 1 year. The possible stabilization seen in our moderate AD patients receiving NBM-DBS thus suggests a potential treatment effect. In contrast, NACC patients with a CDR of 3 showed minimal decline, consistent with a floor effect on these cognitive scales, which may account for the lack of measurable benefit from NBM-DBS in our corresponding severe AD subgroup (Supplementary Figure 2).

### 3.2 Psychobehavioral symptoms and functional abilities

**Figure 2:**
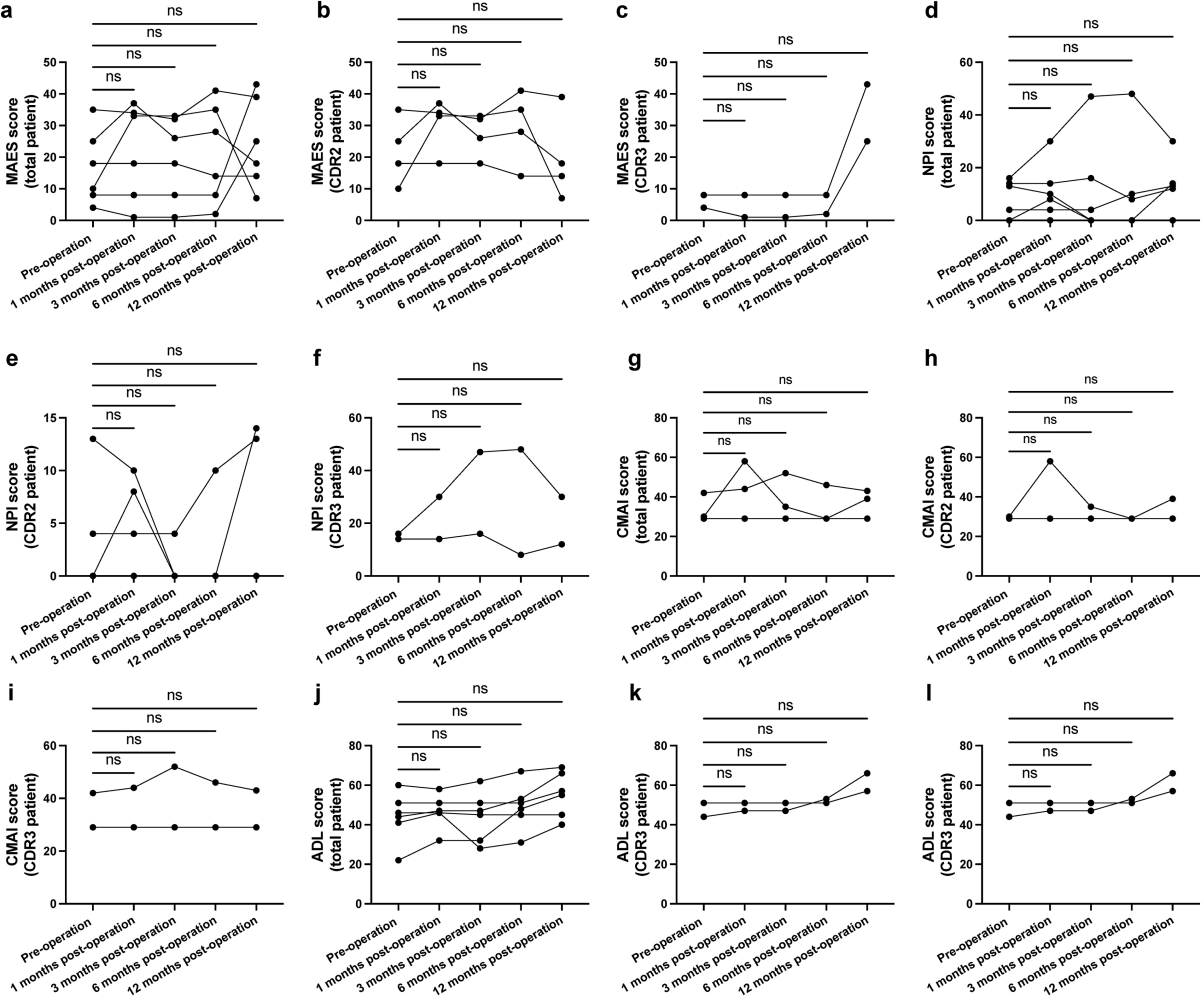
Psychobehavioral symptom scores before and during nucleus basalis of Meynert deep brain stimulation (NBM-DBS). There was no significant change in modified apathy estimate scale (MAES) [**a–c**; **a**: *F*_(1.289, 6.445)_ = 0.4305, *p* = 0.5842; **b**: *F*_(1.501, 4.502)_ = 1.673, *p* = 0.2763; **c**: *F*_(1.000, 1.000)_ = 22.25, *p* = 0.1330], Neuropsychiatric Inventory (NPI) [**d–f**; **d**: *F*_(1.349, 6.744)_ = 0.2670, *p* = 0.6899; **e**: *F*_(2.108, 6.325)_ = 1.103, *p* = 0.3921; **e**: *F*_(2.108, 6.325)_ = 1.103, *p* = 0.3921; **f**: *F*_(1.000, 1.000)_ = 0.7717, *p* = 0.5411], Cohen-Mansfield agitation inventory (CMAI) [**g–i**; **g**: *F*_(1.199, 5.996)_ = 0.8917, *p* = 0.4025; **h**: *F*_(1.000, 3.000)_ = 1.000, *p* = 0.3910; **i**: *F*_(1.000, 1.000)_ = 1.000, *p* = 0.5000], or Activity of Daily Living Scale (ADL) [**j–l**; **j**: *F*_(1.476, 7.379)_ = 3.124, *p* = 0.1113; **k**: *F*_(1.393, 4.178)_ = 1.224, *p* = 0.3553; **l**: *F*_(1.000, 1.000)_ = 3.152, *p* = 0.3265] during treatment. NS, not significant.

For the entire cohort, there was no significant difference in psychobehavioral symptoms as measured by the MAES, NPI, and CMAI at any time point post-operation compared to preoperative baseline. Furthermore, there was no significant change in moderate and severe subgroups. However, there was a numeric increase in the MAES for patients with severe AD at 12 months post-operation compared to baseline (*p* = 0.16) (Figures 2a–i).

### 3.3 Daily living ability measurement

Activities of daily living score did not differ at 1-year compared to preoperative baseline in either moderate or severe AD groups (Figures 2j–l).

### 3.4 Change in inflammation during NBM-DBS

**Figure 3:**
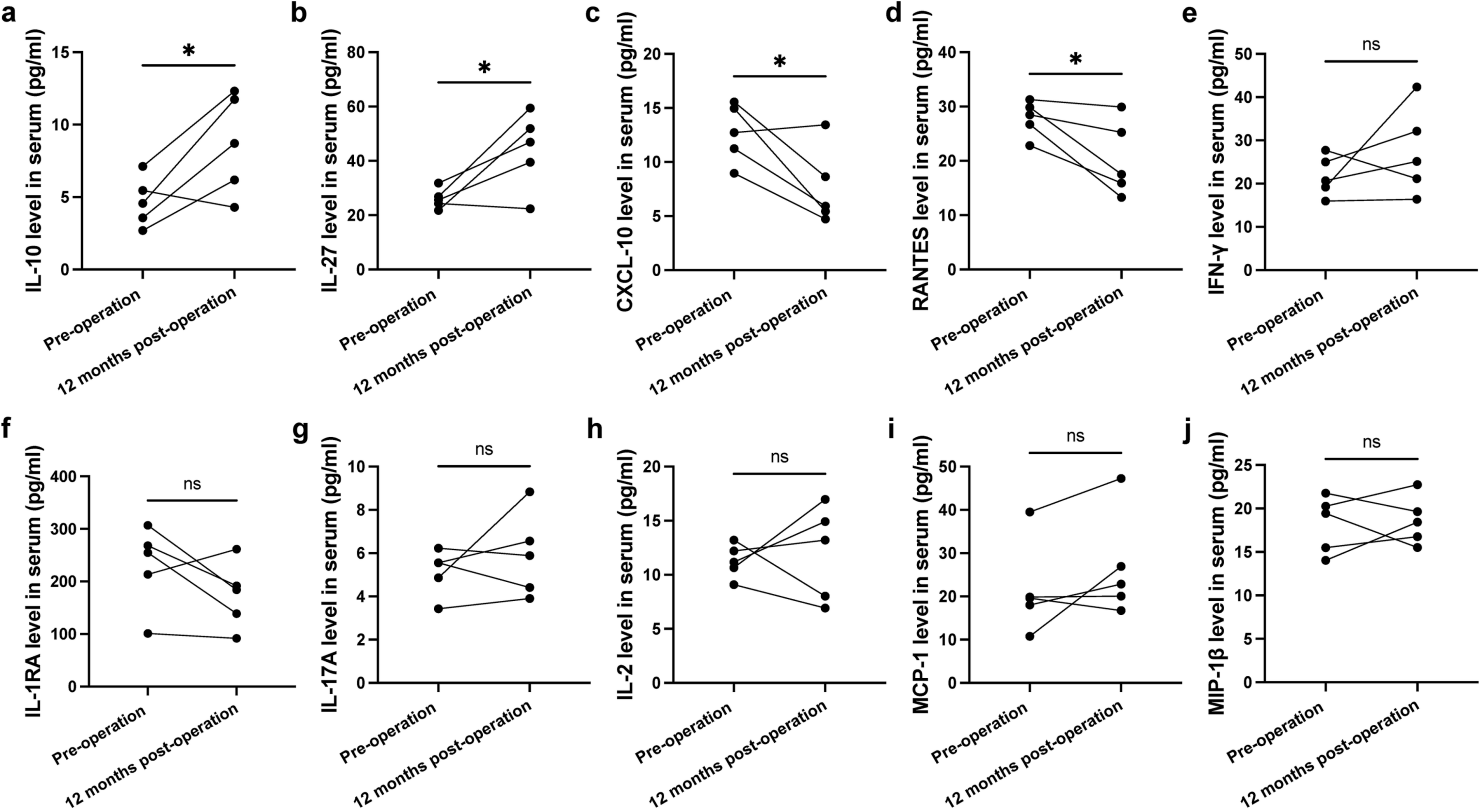
Changes in serum cytokines and chemokines during NBM-DBS treatment. **(a–d)** Serum concentrations of IL-10 **(a)** and IL-27 **(b)** were upregulated during treatment, while serum concentrations of CXCL10 **(c)** and RANTES **(d)** were downregulated. **(e–j)** In contrast, serum concentrations of IFN-γ **(e)**, IL-2 **(f)**, IL-17A **(g)**, IL-1RA **(h)**, CCL-11 **(i)**, and MCP-1β **(j)** remained unchanged. *p < 0.05; ns, not significant.

Serum analysis from five patients revealed that 12 months of NBM-DBS treatment was associated with a significant shift in the inflammatory profile. We observed a marked increase in the anti-inflammatory cytokines IL-10 and IL-27, concurrent with a significant decrease in the pro-inflammatory chemokines CXCL10 and RANTES. Levels of other inflammatory mediators, including IFN-γ, IL-2, IL-17A, IL-1RA, CCL-11, and MCP-1β, remained unchanged (Figure 3).

## 4 Discussion

This study provides two principal insights into the application of NBM-DBS for AD. First, we demonstrate a differential treatment response contingent upon disease severity, with patients in the moderate stage exhibiting possible stabilization in specific cognitive domains, while those with severe AD derived no comparable benefit. Second, we present novel evidence suggesting that the clinical effects of NBM-DBS may be mediated, at least in part, through the modulation of systemic inflammation, as reflected by a shift in serum biomarkers toward an anti-inflammatory state.

Turnbull et al. (15) conducted what is believed to be the first clinical trial exploring DBS for AD treatment. Although the study concluded that DBS lacked a definitive therapeutic effect, it preserved cortical glucose metabolic levels over a 9-month follow-up. The NBM is the primary source of cortical acetylcholine projections, which are essential for normal cognitive function. Furthermore, tau pathology observed in the NBM during AD progression (16), suggests that neuroprotective interventions targeting this region may slow cognitive decline. Beyond anti-inflammatory and metabolic effects, NBM-DBS has been shown to promote increased cortical thickness on T1-weighted MRI, which positively correlated with preserved MMSE scores at 6 and 12 months post-surgery (17), indicating potential long-term neuroprotective efficacy. Kuhn et al. (4) found that bilateral low-frequency NBM-DBS increased the ADAS-cog score by 3 points at 12 months, while the MMSE score decreased by only 0.5 points—substantially less than the natural decline of 1.84 points predicted by regression models. Additionally, Xu et al. reported that NBM-DBS significantly alleviated neuropsychiatric symptoms and sleep disturbances in patients with severe AD (18).

Our finding of substantial possible cognitive stabilization at 12 months in patients with moderate AD aligns with Kuhn et al. (4). In contrast, Xu et al. (18) reported no such benefit, a discrepancy likely attributable to differences in disease stage, as severe AD appeared less responsive to NBM-DBS in our study. By incorporating a public dataset for comparison, we found that moderate AD patients typically show significant cognitive decline, which was blocked by NBM-DBS. Conversely, severe AD patients showed minimal change in cognitive scores, potentially due to a floor effect, and NBM-DBS did not influence their outcomes. Interestingly, BNT scores showed opposite trends in the two patient groups. Based on previous research and our findings, patients with less severe symptoms may derive greater benefit from NBM-DBS. Furthermore, while others have reported that NBM-DBS alleviates neuropsychiatric symptoms (18), we found no such effects. This discrepancy may also be attributed to differences in disease stage and baseline neuropsychiatric symptoms, warranting further investigation. Psychobehavioral symptoms and daily living ability were similar between the two groups 1 year after baseline. Unfortunately, the public dataset did not include these specific scales, preventing a direct comparison of outcomes between DBS patients and natural history controls.

Several recent studies have explored the mechanisms of NBM-DBS. In the APP/PS1 mouse model, NBM-DBS reduced Aβ deposition and neuronal apoptosis while improving performance in spatial memory tasks (19), further supporting its neuroprotective potential. Hotta et al. (20) demonstrated that NBM stimulation in adult rats promoted the secretion of nerve growth factor (NGF), essential for neuronal health. Kuhn et al. (4) also reported significant improvements in hippocampal-amygdalar metabolism following NBM-DBS, consistent with preserved neuronal function. Our previous research revealed that the connectivity between the hippocampal and frontoparietal networks was enhanced in the DBS-on state compared to the DBS-off state (6). Additionally, increased functional connectivity between the parahippocampal gyrus and the parietal cortex was associated with cognitive improvement (6). Collectively, these findings suggested that NBM-DBS may exert therapeutic effects through a combination of neuroprotection and enhanced functional connectivity within key brain networks.

Chronic inflammation is a central driver of neurodegeneration in AD, orchestrated by interactions among microglia, astrocytes, and neurons (21). Persistent cytokine signaling creates a self-sustaining cycle of immune activation and neuronal damage (22). Systemic inflammatory mediators can signal to the brain via neural and humoral pathways, exacerbating pathology (23). Notably, IL-10 deficiency activates microglia and promotes tau hyperphosphorylation (24).

Elevated blood levels of CXCL10 and RANTES have been consistently reported in AD patients (25–27), with CXCL10 localizing around amyloid plaques in mouse models (25) and RANTES mobilizing peripheral immune cells via the CCR5 axis (28). Acetylcholinesterase inhibitors reduce RANTES expression—an effect reversed by Aβ overexpression (28, 29). Our finding that NBM-DBS elevates IL-10/IL-27 and suppresses CXCL10/RANTES suggests it may break this inflammatory cycle, potentially explaining cognitive preservation in early-stage patients. To our knowledge, this is the first study to link NBM-DBS with systemic immunomodulation in AD.

This study also had several limitations. Only six patients completed follow-up, limiting statistical power. However, this reflects the exploratory and costly nature of NBM-DBS, and similar sample sizes are common in pioneering DBS trials (4, 18). A control group was not feasible for ethical and practical reasons; instead, we benchmarked our results against public natural history data (NACC). CSF samples—which better reflect central inflammation—were not collected due to invasiveness concerns, though serum biomarkers remain informative given that inflammatory molecules can cross or signal across the blood–brain barrier (23).

## 5 Conclusion

Bilateral NBM-DBS may be associated with stabilize cognitive function in moderate—but not severe—Alzheimer’s disease, possibly by attenuating inflammatory signaling. In the present study, we observed the system inflammation was affected by DBS. Given that DBS acts directly on nuclei within the brain, we hypothesize that neural immunity is also modulated. Future studies should focus on optimizing stimulation parameters and identifying predictors of clinical response to refine patient selection.

## Data Availability

The datasets presented in this study can be found in online repositories. The names of the repository/repositories and accession number(s) can be found in the article/Supplementary material.

## Footnotes

1
https://naccdata.org
