# Supplementary material for: Clinical outcomes and anti-inflammatory mechanisms of nucleus basalis of Meynert deep brain stimulation in Alzheimer’s disease
Source: Front Neurol. 2026 Apr 22;17:1773910. doi: 10.3389/fneur.2026.1773910 (PMC13143682; doi:10.3389/fneur.2026.1773910)
Supplement: Supplementary file 1 [file Supplementary_file_1.docx]

**Supplementary File**


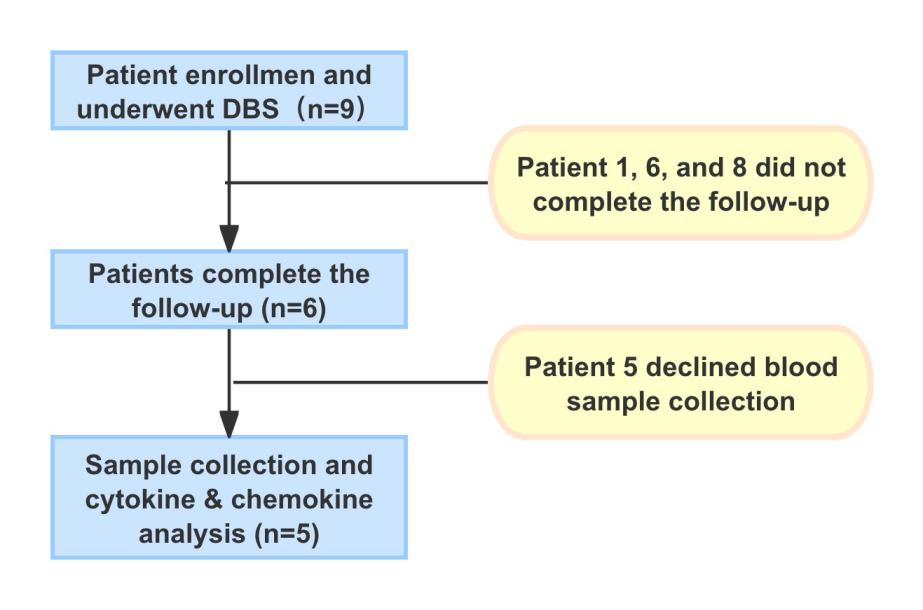


**Supplementary Figure 1:** Flow diagram of this study. From the initial enrollment of nine patients, six completed the full 12-month follow-up; three were lost primarily due to constraints related to the COVID-19 pandemic. Blood samples from a final total of 5 patients were used for the final analysis. Patient 1, 6, and 8 were scheduled for visits in January 2020, October 2020, and July 2021, respectively.


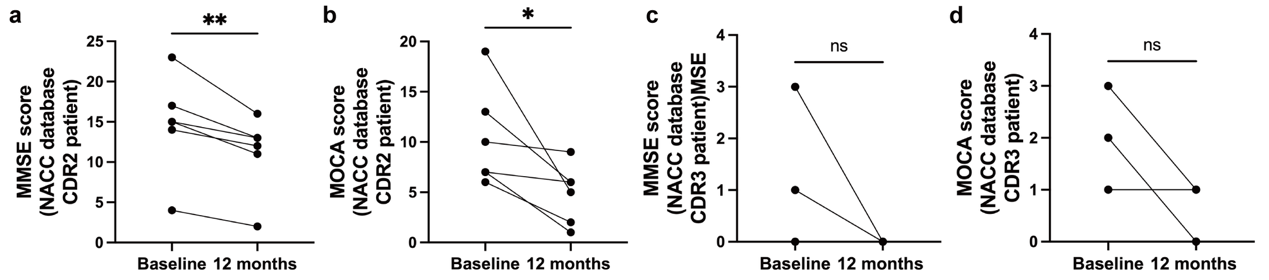


**Supplementary Figure 2:** Change in cognitive scales of patients from public data set. For patients with CDR 2, MMSE (a) and MoCA (b) score was significantly decreased 1-year after baseline. However, for patients with CDR 3, it is not changed in these scores (c, d). **P* < 0.05; ns, not significant.
